# The Shockwè trap: a human-baited exposure-free device for surveillance and behaviour studies of anthropophilic vectors

**DOI:** 10.12688/wellcomeopenres.19963.1

**Published:** 2023-10-12

**Authors:** Ayubo Kampango, Thomas A. Smith, Ana Paula Abílio, Elias Alberto Machoe, Júlio Francisco Matusse, João Pinto, Philip J. McCall

**Affiliations:** 1 Vectors Study Unit, Instituto Nacional de Saúde, Maputo Province, Vila de Marracuene, Mozambique; 2 Department of Epidemiology and Public Health, Swiss Tropical and Public Health Institute, Allschwil, Switzerland; 3 University of Basel, Basel, Switzerland; 4 Global Health and Tropical Medicine, Institute of Hygiene and Tropical Medicine, Lisbon, Portugal; 5 Department of Vector Biology, Liverpool School of Tropical Medicine, Liverpool, UK

**Keywords:** Mosquitoes, traps, host-seeking, Shockwè trap, HLC, trapping efficiency, indoor, outdoor

## Abstract

**Background:** The human biting rate (MBR) and entomological inoculation rate (EIR) are common parameters routinely used to measure the risk of malaria transmission. Both parameters can be estimated using human landing catches (HLC). Although it is considered the gold-standard, HLC puts collectors at higher risk of infection with mosquito-transmitted pathogens.

**Methods:** A novel exposure-free host-seeking mosquito electrocution trap, the Shockwè trap (SHK), was developed and its efficiency for monitoring mosquito community composition and abundance was compared with human landing catches (HLC) as the gold-standard. Field experiments were performed in Massavasse village, southern Mozambique. Simultaneous indoor and outdoor collections of nocturnal host-seeking mosquitoes were carried out using the SHK and HLC methods. The relative sampling efficiency of SHK was estimated as the ratio of the numbers of mosquitoes caught in SHK compared HLC. Proportionality and density-dependence between SHK and HLC catches were estimated by mean of Bayesian regression approaches.

**Results:** A total of 69,758 and 27,359 host-seeking mosquitoes comprising nineteen species and four genera, were collected by HLC and SHK respectively. In general, SHK and HLC sampled similar numbers of mosquito species, with the exceptions of the least common species *Aedes sudanensis*, *Ae. subargenteus*, and *Coquillettidia versicolor* that were caught only by HLC. The relative sampling efficiency and proportionality between SHK and matched HLC catches varied greatly between species and collection site. However, all mosquitoes collected by SHK were unfed, confirming the Shockwè trap design’s performance and reliability as a successful mosquito exposure free sampling approach.

**Conclusions:** Results demonstrate that SHK is a safe and reliable human-exposure free device for monitoring the occurrence of a wide range of mosquito, including major malaria and arboviruses vector species. However, improvements are needed to increase its sampling efficiency for less abundant mosquito species.

## Introduction

The magnitude of a human host’s exposure to biting by the mosquito vector is a key parameter in epidemiological studies of malaria. This biting exposure is still determined using the human landing catch (HLC), a method first introduced in the 1930s ^1^, but that continues to be widely used and accepted as the gold standard method for measuring biting exposure. A simple procedure requiring minimal equipment, HLC also provides a reliable method for measuring diurnal patterns of vector activity, and for identifying body regions most frequently contacted or preferentially bitten by a particular species ^2^. However, HLC has serious operational drawbacks: it is physically demanding and its accuracy greatly depends on the individual collector’s stamina, honesty, experience, their inherent attractiveness to mosquitoes ^3– 5^ and their ability to catch them, especially at high biting rates ^6^. Collectors may face a higher risk of disease transmission by the mosquitoes being studied or by other species, particularly the mosquito vectors of arboviruses against which there are no effective vaccines, like dengue, Zika and chikungunya. This may not always be true, because, at least in some circumstances, there is good evidence that, with malaria, there is negligible risk to HLC collectors ^7, 8^. Hence, a long-standing goal for medical entomologists and vector epidemiologists has been the development of a sampling device that can accurately reproduce the entomological indicator obtained with HLC without posing risk to collectors.

Typically, traps for sampling host-seeking anthropophilic mosquito vectors either incorporate a live human bait or are designed to incorporate a human host mimic assembled from visual or olfactory cues. The yield of traps with synthetic baits can be dramatically improved using carbon dioxide, but the expense and logistics of using and controlling this highly volatile mosquito stimulant/attractant, has severely limited its effective use in the majority of routine applications to date. Moreover, the physiological effect of several synthetic odour compound blends remains poorly defined, as the level of attractiveness can dramatically vary depending on the vector species ^9, 10^. Moreover, some commonly used attractant blends, as well as CO _2_ can function as repellents at certain concentrations ^11^.

A wide range of traps exist, and many innovative designs continue to be developed today ^12– 20^. A number of studies have evaluated and compared some of the more popular traps ^21– 24^, and although there is no single trap design that is consistently reported as being an improvement on HLC, the simple, compact, and relatively inexpensive CDC light trap is very widely used for sampling and surveillance of nocturnally active mosquitoes worldwide. A major advantage of light traps is that sampling can be conducted across large areas in short time periods, with minimal effort. However, light traps also have limitations. For example, many species of vector mosquitoes routinely sampled by light traps do not naturally orient to light, raising questions over what the mosquitoes are responding to the trap's light or whether they are simply being captured passively as they drift close to the trap’s fan. Moreover, light trap collections are greatly compromised by environmental conditions, particularly moonlight ^6, 25, 26^, to an extent where outdoor collections are not directly comparable with indoor collections performed simultaneously ^6, 27^.

Here we report on the development and field testing of a novel trap for collecting mosquitoes attracted to a sleeping human bait, a device with potential for use indoors and outdoors. The trap exploits the knowledge that *Anopheles gambiae s.l.* and other mosquitoes preferentially descend onto a supine human host ^28, 29^, the sleeping position presented to hungry host seeking female anophelines and other nocturnally active mosquitoes by the vast majority of humans. The trap can be used by a single person (or with another source of attractant), who remains free from exposure to mosquito bites for the entire sampling period, while the mosquito responds to natural olfactory and physical stimulants produced by the host.

## Methods

### Ethical considerations

The study received ethical approval from the Research Ethics Committee at Liverpool School of Tropical Medicine (Research Protocol 14.055, *Host Location by Exophagic African Malaria Vector*s) on 28 ^th^ January 2015 and from the *Comité Nacional de Bioética para Saúde* (CNBS) of the Ministry of Health of Mozambique (MISAU), Ref: 208/CNBS/15, on 22 ^nd^ July 2015.

To minimise the risk of infection, all volunteers (for both HLC and SHK) during the field studies were provided with anti-malaria prophylaxis, Fansidar ^®^ (Sulfadoxine-Pyrimethamine), in accordance with the recommendations of the Mozambique National Malaria Control Program at the time the study was undertaken. A recent study showed that this practice reduced risk of malaria infection in HLC volunteers by 96.6% compared to a matched control group of individuals not involved in the study but living in the same locality ^7^.

### Consent

Written informed consent was provided by all volunteers participating in the study, by signing a form that was co-signed by the senior investigator.

### Description of study site

**Figure 1.**
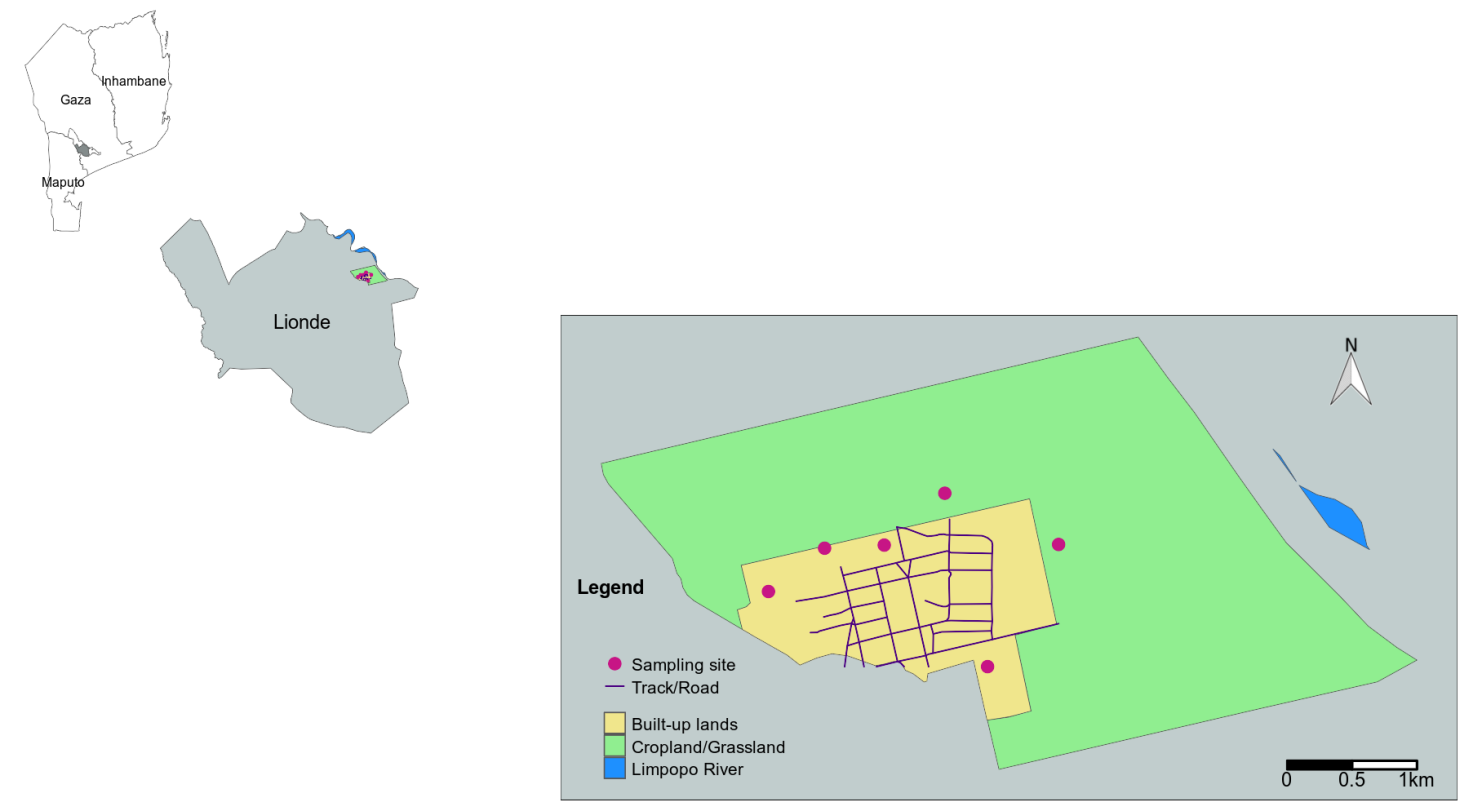
Location of Gaza Province, southern Mozambique showing the location of Lionde administrative post where the study site at Massavasse is situated.

The study was conducted during the wet season February to April 2016 in Massavasse village (-24.624839° S; 33.111787° E). The village is located in Chókwè district, southwest Gaza Province, Mozambique) ^30^ ( Figure 1). The mean temperature in this period typically ranges from 25–34°C, and this is also the season experiencing most of the annual rainfall of 600 mm. *Anopheles arabiensis*, *An. funestus*, *An. gambiae s.s*, *An. tenebrosus*, *An. pharoensis* and *An. ziemanni* are the most common malaria vectors in the village. Several other mosquito species within the genera *Culex* and *Aedes* known to be vectors of arboviral diseases have been also found in the village ^30, 31^.

### The Shockwè trap

**Figure 2.**
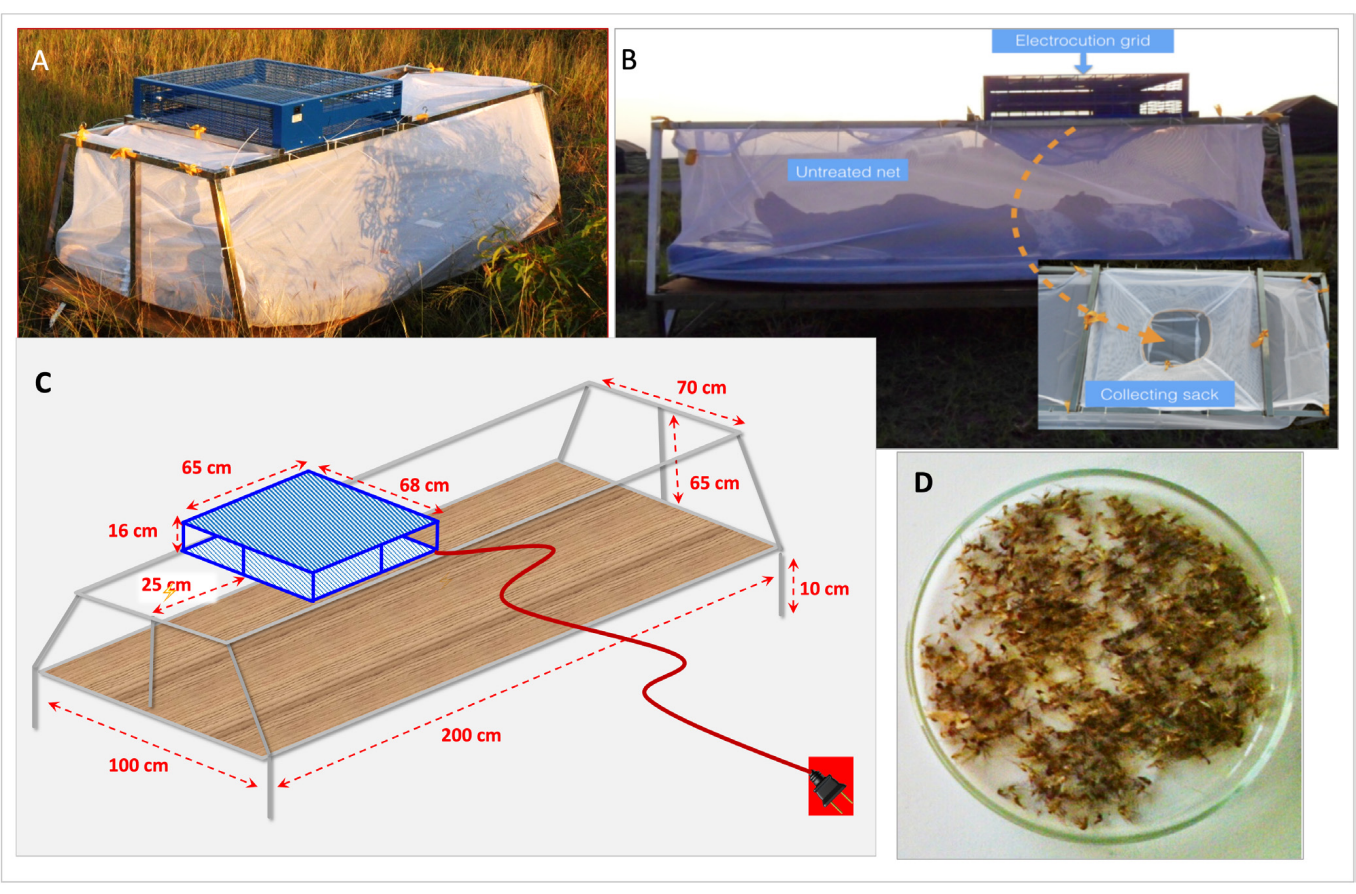
The Shockwè trap (SHK) prototype showing ( **A**) SHK as deployed outdoors in use in the field; ( **B**) a side view of a volunteer within the trap and a top view of the collection sack or bag, which, when in use, is positioned beneath the electrocution grid; ( **C**) the dimensions and structure forming the trap frame, and ( **D**) a typical sample of mosquitoes from one SHK trap on one night collection using in Massavasse village.

The Shockwè trap (SHK) ( Figure 2) comprised a metal frame, 200 cm in length, 100 cm wide at the base, 70 cm wide at the top and 65 cm in height from the trap base to the roof (equivalent to the maximum flight height reported for several *Anopheles* species ^32, 33^). The base of the trap was 10 cm from the floor, compatible with the reported flight height of several mosquito species ^34– 36^. A modified insect electrocution grid device (Bower Products, London;), delivering 3,800 volts at 9 milliamperes (input of 230 volts) at the electric grid, was deployed on top of the frame. The device was fully compliant with EU safety specifications for use (BS EN 60335F2F59 and the European EMC directive). The electrocution grid measured approximately, 65 × 68 cm (16 cm deep) and the area of the active electric net was 493 × 500 mm. A grounded outer aluminium mesh of a size that allowed mosquito entry but prevented any human body parts from contacting the grid protected the live wires. The electric grid was positioned on the frame above the head and torso region of a supine adult lying beneath, as this is where the majority of both *Anopheles* (and *Culex spp.*) approach the host at the top surface of the net ^28, 37^ ( Figure 2). The human bait slept below the electrocution grid inside an untreated bed net trap which had been modified to function as a mosquito collecting sack ( Figure 2). The electricity supply incorporated a circuit breaker (a residual current device or RCD), and in the event of a short circuit, the circuit would cut out instantly, significantly reducing the risk of serious injury. In the field, the trap was powered by a 7.5 kW 4-stroke Ryobi™ generator, providing an uninterrupted alternate electric current (AC) supply to the traps for up to 11.50 hours without refuelling.

### Experimental design

The experiments were conducted in five of Massavasse’s six neighbourhoods. A series of paired indoor and outdoor collections were conducted in six selected sentinel sites located at the five selected neighbourhoods ( Figure 2). In each site, four experienced adult collectors were randomly assigned to perform paired mosquito collections indoor and outdoor by mean of HLC and using SHK trap. All collectors were trained adult males, above 18 years old. Indoor collections were made using a purpose-built portable experimental hut, described previously ^30^. The relative positions of the paired collectors performing HLC and SHK were located 12 to 20 metres apart to prevent any possible interference between them. Collection was performed from 19:00 to 05:00 hrs, and collectors performed hourly rotation between collection methods to minimize the effect of different attractiveness of different collectors or their catching ability on the quality of collected data.

### Sample processing and analysis

Collected mosquito samples were immobilised within paper cups inside a refrigerator (approx. 4°C) for 20 to 30 minutes. Anopheline mosquito species were identified morphologically to species or species complex level (e.g., *An. gambiae s.l.*) using the taxonomic keys of Gillies & De Meillon ^38^ and Gillies & Coetzee ^39^. Culicine mosquitoes were identified using taxonomic keys of Edwards ^40^, Jupp ^41^, Harbach ^42^, and Service ^43^. Members of the major vector species complexes, such as *An. gambiae s.l* or vector species groups (e.g., *An. funestus s.l*) were identified to species level by molecular analysis (PCR). The protocols of Scott *et al.* ^44^ and Koekemoer *et al.* ^45^ were used to detect species-specific single nucleotide polymorphisms (SNPs) in the intergenic spacer region (IGS) and internal transcribed spacer region 2 (ITS2) of ribosomal DNA (rDNA) genes for identifying members of *An. gambiae* complex and *An. funestus* group, respectively ^44, 45^.

### DNA extraction

Whole mosquitoes were transferred into 1.5 ml Eppendorf tubes and submerged with 200 µl of Tris-EDTA buffer solution at pH 7.8 (Sigma-Aldrich ^®^, USA). The samples were then macerated until homogenize and incubated at 94°C for 12 minutes, after which the homogenized were centrifuged at 13,000 rpm for 4 minutes for deposition of fragments of exoskeleton tissues. Then, 100 µL of the supernatant (solution containing DNA) were transferred to a new pre-coded Eppendorf tube, and store at -20°C for further analysis.

### DNA amplification

Amplification of extracted DNA sample was performed by mean of conventional PCR. The primers and reagents for master mix used are indicated in Table 1. The primers were obtained from Bei Resources ( https://www.beiresources.org). 1.2 µL of extracted DNA were added to solutions containing the master mix. For identification of members of *An. gambiae* complex, PCR reactions were run with the following condition: one initial denaturing cycle at 94°C for 2 minutes. Thirty denaturing, annealing and extension cycles at 94°C, 50°C and 72°C, respectively, all for thirty seconds, a final extension cycle at 72°C for 5 minutes. Reaction products were hold at 4°C inside thermocycle (Biometra ^®^, USA) before visualization using electrophorese gel ^44^, mixed with 10ul of SybrSafe (ThermoFisher ^TM^, USA). For *An. funestus* group, the following programmer were used: one initial denaturing cycle at 94°C for 2 minutes. Thirty denaturing, annealing and extension cycles at 94°C, 45°C and 72°C, respectively, all for thirty-six seconds, and a final extension cycle at 72°C for 5 minutes, and a holding cycle at 4°C for indefinite time until visualization ^45^. For *An. gambiae s.l* members, the reactions creates DNA fragments of 464Pb ( *An. merus*, ME), 390Pb ( *An. gambiae*, GA), 315Pb ( *An. arabiensis*, AR), 153Pb ( *An. quadrianulatus*, QD), 153Pb ( *An. quadrianulatus*, QDA). Likewise, for *An. funestus* group the primers create DNA fragments of 600Pb ( *An. vaneedine*, VAN), 464Pb ( *An. funestus s.s*, FUN), 4111Pb ( *An. rivolurum*, RIV), 4111Pb ( *An. rivolurum* Like), 252 Pb ( *An. parensis*, PAR) and 146Pb, 166Pb ( *An. leesoni*, LEES) ^44^.

**Table 1.** Reagents and primers used for preparation of master mixes for molecular identification of member of *An. gambiae* complex ^44^ and *An. funestus* group ^45^.

Species	Reagents	Qty
*Anopheles gambiae s.l.*	Sterile H20	1.25 μL
	Taq 5x PCR Buffer	1.25 μL
	dNTP (2.0 mM mix)	0.5 μL
	Mgcl2 (25mM)	0.5 μL
	UN (F, 25 pmol/μl) [GTG TGC CCC TTC CTC GAT GT]	1 μL
	AR (R, 25 pmol/μl) [AAG TGT CCT TCT CCA TCC TA]	
	GA (R, 25 pmol/μl) [CTG GTT TGG TCG GCA CGT TT]	
	ME (R, 25 pmol/μl) [TGA CCA ACC CAC TCC CTT GA]	
	QD (R, 25 pmol/μl) [CAG ACC AAG ATG GTT AGT AT] OR	
	QDA (R, 25 pmol/µl) [CAT AAT GAG TGC ACA GCA TA]	
	GoTaq DNA polymerase (5 U/ul)	0.1 μL
*Anopheles funestus s.l.*	Sterile H20	1.25 μL
	Taq 5x PCR Buffer	1 μL
	dNTP (2.0 mM mix)	0.75 μL
	Mgcl2 (25mM)	1 μL
	UV (F, 33 pmol/μl) [TGT GAA CTG CAG GAC ACA T]	2.9 μL
	FUN (R, 33 pmol/μl) [GCA TCG ATG GGT TAA TCA TG]	
	VAN (R, 33 pmol/μl) [TGT CGA CTT GGT AGC CGA AC]	
	RIV (R, 33 pmol/μl) [CAA GCC GTT CGA CCC TGA TT]	
	PAR (R, 33 pmol/μl) [TGC GGT CCC AAG CTA GGT TC]	
	RIVLIKE (R, 33 pmol/μl)[CCG CCT CCC GTG GAG TGG GGG]	
	LEES (R, 33 pmol/μl) [TAC ACG GGC GCC ATG TAG TT]	
	GoTaq DNA polymerase (5 U/ul)	0.1 μL

### Statistical analysis

***Estimation of sampling efficiency.*** Commonly used methods for analyzing agreement between measures ^46^ are not suitable for mosquito count data as counts are usually very skewed and highly over dispersed. In addition, the usual approach of log transforming counts to reduce the degree of skewness is not applicable because of the frequency of counts of zero ^47^. However, mosquito counts in candidate sampling devices can be ranked, and agreement with matched HLC quantified using rank concordance correlation coefficients (CCC). CCC statistics quantify the variability in the data but do not give any indication of whether the novel method is collecting information that is comparable with the standard approach. Estimates of the trapping efficiency can be computed by taking the ratios of total numbers of mosquitoes caught by the different methods, but these are very sensitive to outliers in the counts, either of the novel or the standard methods ^47^.

In view of these constraints, to determine SHK trap sampling efficiency relative to HLC, we used a less outlier-sensitive regression-based approach, that had been originally proposed by Hii *et al*., ^48^, and was adopted subsequently by other authors ^17, 27, 49^. Comparisons were made between indoor HLC, outdoor HLC, indoor SHK trap and outdoor SHK trap. Strata were defined by combinations of sampling locations and days. The number of strata included in the analysis varied by mosquito species taxon.

The following linear statistical model was used to estimate the sampling efficiencies of SHK relative to HLC,

$$E(y_{i,m})=\alpha_{m}E(x_{i,m})\;(1)$$

where: *E*( *y _i_*) is the expected number of mosquitoes of a given taxon caught using method *m* in stratum *i;* *E*( *x _i_*) is the expected number of mosquitoes of the same taxon caught using an indoor HLC method in the same stratum *i*; *α _m_* is the relative sampling efficacy for method *m*, compared to indoor HLC for which the value is set to unity. The underlying mosquito density *E*( *x _i_*) is assumed to have a log-normal distribution, *i.e*. $ln\;(E(x_{i,m}))∼Normal(\mu_{s},\sigma_{s}^{2}),$ Poisson errors were assumed in the observed numbers of mosquitoes caught by each methods so that: *x _i_* ~ *Poisson*( *E*( *x _i,m_*)) and: *y _i,m_* ~ *Poisson*( *E*( *y _i,m_*)) and the model therefore assumes the distribution of the numbers of mosquitoes caught by any method to be a log-normal mixture of Poisson distributions.

To examine whether the trapping efficiency varied with the average mosquito density, the following extended model was also fitted:

$$E(y_{i,m})=(\alpha {'}_{m}E{(x_{i,m}))}^{\gamma_{m}}\;(2)$$

where *γ _m_* is an exponent corresponding to method *m.*

If the different methods are sampling the same fraction of the mosquito population, then the fitted line for model (1) should be close to that for model (2). Equivalently, the 95% credible intervals for *y _m_* should overlap with unity. A value of *y _m_* that is different from unity indicates a lack of proportionality between the mosquito sampling methods. In addition, *α' _m_* will differ from *α _m_* if *γ _m_* is different from unity ^48^. The model was fitted using a Bayesian Markov chain Monte Carlo algorithm in rjags v. 4 -14 ^50^.

## Results

### Abundance and composition of mosquito collections

A total of 35 nights of paired HLC and SHK collections yielded a total catch of 97,117 mosquitoes comprising twenty-three species in five genera ( Table 2). Sampling with HLC and SHK yielded 69,758 and 27,359 specimens respectively, with *Culex* and *Anopheles* being the most common genera sampled by both methods.

**Table 2.** Relative abundance and composition of mosquito collections by each sampling methods: Human landing catch (HLC) and Shockwè Trap (SHK); deployed indoors or outdoors in Massavasse village.

Species	HLC		SHK	
	Indoor	Outdoor	Indoor	Outdoor
*Anopheles funestus s.l*	40	29	11	1
*Anopheles gambiae s.l*	3,403	4,437	725	147
*Anopheles pharoensis*	28	1,950	126	338
*Anopheles tenebrosus*	25	1,023	1	38
*Anopheles ziemanni*	53	1,413	-	65
*Aedes fryeri*	4	73	14	127
*Aedes sudanensis*	1	4	-	-
*Aedes subargenteus*	-	4	-	-
*Culex pipiens*	1,019	4,971	353	275
*Culex poicilipes*	1,219	4,444	177	664
*Culex tritaeniorhynchus*	1,007	7,504	547	1,963
*Culex antennatus*	229	1,625	91	282
*Culex quinquefasciatus*	43	9	33	3
*Culex bitaeniorhynchus*	2	3	1	11
*Culex sitiens*	1	2	-	1
*Coquillettidia aurites*	1	8	4	-
*Coquillettidia versicolor*	-	1	-	-
*Mansonia africana*	1,521	2,594	510	857
*Mansonia uniformis*	11,156	19,912	5,467	14,527

*Anopheles* species comprised *Anopheles gambiae s.l*, *An. pharoensis, An. tenebrosus, An. ziemanni* and *An. funestus*, and accounted for 17.8% (12,401 n = 13,853) of the total (indoor and outdoor) *Anopheles* species catches obtained by HLC and 5.3% (n = 1,452) sampled by SHK. *Culex tritaeniorynchus* was the predominant species amongst eight *Culex* species sampled by either method ( Table 2). These species represented 31.6% (n = 22,078) and 16.1% (n = 4,401) of specimens sampled by HLC and SHK, respectively. The genus *Mansonia* was represented by two species, *Ma. africana* and *Ma. uniformis* which accounted for 50.4% (n = 35,183) and 78.1% (n = 21,361) of all specimens sampled by HLC and SHK, respectively.

Two *Aedes* mosquito species, namely *Ae.* ( *Stegomyia*) *subargenteus* and *Ae* ( *Muscidus*) *sudanensis*, and one *Coquillettidia* species were only collected by HLC ( Table 2). The underlying human landing catch and Shockwè trap raw data can be found here ( https://doi.org/10.17605/OSF.IO/U5A4V) ^51^.

### Molecular identification of species complex/group members

Of 1,052 mosquitoes subjected to molecular analysis, 980 were identified as members of the *An. gambiae* complex and 72 as in the *An. funestus* group ( Table 3). Of the 980 *Anopheles gambiae s.l*, 850 were collected by HLC and 176 by the SHK 701 were identified as *Anopheles arabiensis*, 20 as *An. gambiae s.s*, 68 as *An*. *merus* and 26 as *An*. *quadriannulatus*. Of 31 *Anopheles funestus* group processed, 22 were *An*. *funestus s.s* and 7 were *An*. *parensis.* All the members of *An. gambiae s.l* and *An. funestus* identified by PCR were detected in the subsamples from both SHK and HLC, with the exception of *An*. *merus* and *An*. *parensis*, which were collected only with HLC ( Table 3). A total of 126 and 60 specimens from HLC and SHK respectively, initially identified morphologically as members of *An. gambiae* complex did not amplify.

**Table 3.** Result of PCR analysis on subsamples of *Anopheles gambiae s.l* and *Anopheles funestus s.l* from human landing catch (HLC) and Shockwè Trap (SHK).

Method	Species complex/ group	Molecular ID	Total	
			Indoor	Outdoor
HLC	*Anopheles gambiae s.l*	*Anopheles gambiae s.s.*	2	0
		*Anopheles arabiensis*	334	270
		*Anopheles merus*	40	28
		*Anopheles quadriannulatus*	3	22
	*Anopheles funestus s.l*	*Anopheles funestus s.s.*	10	13
		*Anopheles parensis*	7	0
	*Anopheles gambiae s.l*	Not amplified	24	83
	*Anopheles funestus s.l*		9	10
SHK	*Anopheles gambiae s.l*	*Anopheles gambiae s.s.*	9	9
		*Anopheles arabiensis*	32	65
		*Anopheles merus*	0	0
		*Anopheles quadriannulatus*	1	0
	*Anopheles funestus s.l*	*Anopheles funestus s.s.*	21	0
		*Anopheles parensis*	0	0
	*Anopheles gambiae s.l*	Not amplified	45	14
	*Anopheles funestus s.l*		1	0

### Shockwè trap efficiency

**Figure 3.**
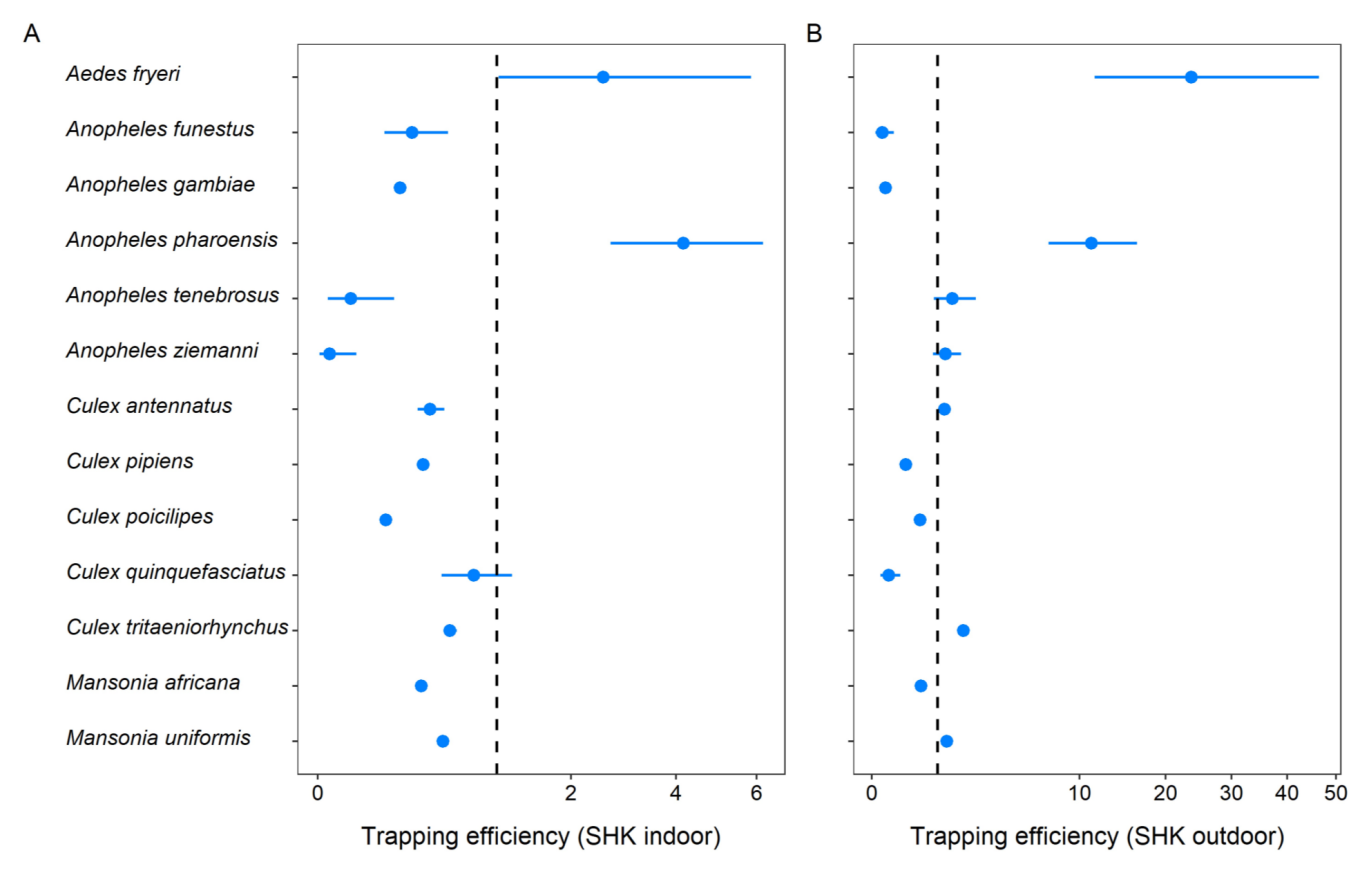
The efficiency of the Shockwè trap (SHK) trap deployed indoors ( **A**) and outdoors ( **B**) in Massavasse village. In each case the vertical dashed line corresponds to a trapping efficiency of 1.0 (indicating nondifference). The error bars are 95% credible intervals.

Rank concordance correlation analyses between HLC and SHK catches are summarized in Table 3. For most species, estimates of CCC suggested moderate association between SHK and matched HLC catches ( Table 4). Regression model analyses indicated that trapping efficiency of SHK varied in function of mosquito species. Indoor and outdoor, the result indicated that SHK was efficient at sampling *Ae. fryeri* and *An. pharoensis* ( Figure 3A, Table 5). In addition, the trap was also efficient in sampling *Cx. bitaeniorhynchus*, *Cx. tritaeniorhynchus* and *Ma. uniformis* outdoor ( Figure 3B; Table 5). The ratio of total caught outdoor to indoor suggests that these mosquito species were primarily exophagic ( Table 5).

**Table 4.** Total mosquitoes caught in Massavasse village, total strata, and concordance correlation coefficients. Indoor human landing catch (HLC) collections were arbitrarily considered as reference for concordance analysis. SHK: Shockwè Trap.

Species	Total collected	Total strata	Rank concordance correlation coefficients		
			HLC	SHK	SHK
			outdoor	indoor	outdoor
*Anopheles funestus*	81	20	0.579	0.531	0.486
*Anopheles gambiae s.l*	8,712	35	0.487	0.488	0.509
*Anopheles pharoensis*	2,442	34	0.551	0.56	0.497
*Anopheles tenebrosus*	1,087	31	0.477	0.454	0.595
*Anopheles ziemanni*	1,531	25	0.534	0.5	0.596
*Aedes fryeri*	218	12	0.331	0.401	0.613
*Aedes sudanensis*	5	5	0	0.5	0.5
*Aedes subargenteus*	-	1	4	-	-
*Culex antennatus*	2,227	16	0.503	0.523	0.546
*Culex bitaeniorhynchus*	17	7	0.746	1	0.5
*Culex pipiens*	6,618	30	0.587	0.503	0.598
*Culex poicilipes*	6,504	35	0.517	0.327	0.492
*Culex quinquefasciatus*	88	14	0.528	0.6	0.595
*Culex sitiens*	4	3	0.25	0.5	1
*Culex tritaeniorhynchus*	11,021	34	0.447	0.425	0.453
*Coquillettidia aurites*	13	7	0.306	0.377	0.5
*Coquillettidia versicolor*	1	1	1	-	-
*Mansonia africana*	5,482	35	0.665	0.345	0.583
*Mansonia uniformis*	51,062	35	0.479	0.449	0.587

**Table 5.** Estimates of trapping efficiency ( *α*) from linear models (95% credible intervals). Indoor human landing catch (HLC) collections were arbitrarily considered as reference in regression models. Only species that occurred on at least ten sampling occasions (strata) were included in the models. SHK: Shockwè Trap; DIC: Deviance information criterion.

Species	HLC outdoor	SHK			DIC linear model	DIC power model
		indoor	outdoor	Ratio (Outdoor: Indoor)		
*Anopheles funestus*	0.72 (0.44, 1.18)	0.27 (0.13, 0.52)	0.02 (0.00, 0.12)	0.09	182.76	175.26
*Anopheles gambiae s.l*	1.31 (1.25, 1.37)	0.21 (0.20, 0.23)	0.04 (0.04, 0.05)	0.2	2577.69	2414.28
*Anopheles pharoensis*	68.28 (49.07, 99.80)	4.40 (3.02, 6.54)	11.82 (8.28, 17.39)	2.69	1734.84	1210.9
*Anopheles tenebrosus*	40.08 (27.66, 62.22)	0.04 (0.00, 0.18)	1.47 (0.89, 2.47)	39.01	419.11	419.13
*Anopheles ziemanni*	26.92 (20.62, 35.96)	0.00 (0.00, 0.05)	1.23 (0.85, 1.78)	277.41	403.86	404.81
*Aedes fryeri*	16.05 (7.35, 37.18)	2.97 (1.14, 7.95)	27.98 (12.85, 65.79)	9.41	202.98	121.1
*Culex antennatus*	7.08 (6.17, 8.14)	0.39 (0.31, 0.50)	1.23 (1.03, 1.46)	3.12	1602.21	1428.78
*Culex pipiens*	4.88 (4.57, 5.21)	0.35 (0.30, 0.39)	0.27 (0.23, 0.31)	0.78	3156.33	2689.15
*Culex poicilipes*	3.65 (3.42, 3.87)	0.15 (0.12, 0.17)	0.54 (0.50, 0.60)	3.75	2158.39	2094.89
*Culex quinquefasciatus*	0.21 (0.09, 0.41)	0.78 (0.50, 1.20)	0.07 (0.02, 0.19)	0.09	201.9	194.32
*Culex tritaeniorhynchus*	7.45 (7.01, 7.97)	0.54 (0.49, 0.60)	1.95 (1.81, 2.10)	3.59	4543.6	4403.45
*Mansonia africana*	1.71 (1.60, 1.83)	0.34 (0.30, 0.37)	0.56 (0.52, 0.62)	1.68	2778.16	2638.87
*Mansonia uniformis*	1.79 (1.74, 1.83)	0.49 (0.47, 0.51)	1.30 (1.27, 1.33)	2.66	14708.76	13024.37

**Figure 4.**
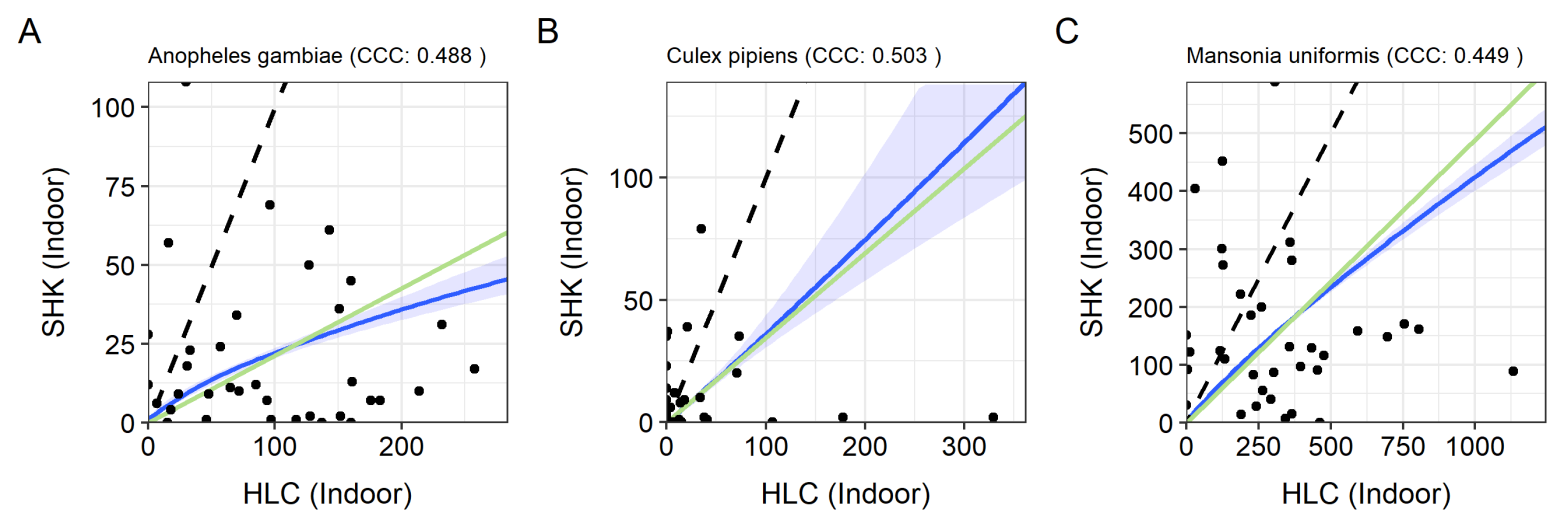
Proportionality between Shockwè trap (SHK) and human landing catch (HLC) indoor catches of *Anopheles gambiae s.l*, *Culex pipiens* and *Ma. uniformis*. In each case, the dashed diagonal line corresponds to a 1:1 ratio of the two methods (trapping efficiency 1.0). The green straight lines are the fitted lines from the linear models. The blue curves correspond to the fitted lines from the power model and the blue shading to the 95% credible interval around it.

Regression model parameters showing the degree of proportionality between SHK and HLC catches, and density-dependency of SHK performance are depicted in Table 6. For most of species, regression models indicated that the number of mosquitoes collected by SHK were density dependent, the 95% credible interval for the exponent ( *γ _m_*) did not overlapped with unity ( Table 6). The exception was observed with *An. gambiae s.l* (indoor), *Cx pipiens s.l* (indoor) and *Ma. uniformis* (indoor) ( Table 6, Figure 4). For other species that SHK and HLC collections were at some point proportional, such as *An. funestus s.l* (indoor and outdoor), *An. tenebrosus* (indoor and outdoor), *An. ziemanni* (indoor and outdoor), *Ae. fryeri* (indoor), the large 95% credible interval indicates huge uncertainty ( Table 6).

**Table 6.** Parameter estimates from power models (95% credible intervals) applied to investigate density-dependence in sampling performance of Shockwè trap (SHK) compared with human landing catch (HLC) *.* Indoor HLC collections were arbitrarily considered as reference in regression models. Only species that occurred on at least ten sampling occasions (strata) were included in the models.

Species	HLC		SHK			
	HLC outdoor		Indoor		Outdoor	
	*α'*	γ	*α'*	γ	*α'*	γ
*Anopheles funestus*	4.71 (0.46, 211.96)	0.23 (0.02, 0.58)	0.25 (0.14, 0.45)	1.37 (0.71, 2.31)	0.01 (0.00, 0.11)	0.89 (0.30, 2.01)
*Anopheles gambiae s.l*	0.26 (0.20, 0.33)	1.43 (1.31, 1.54)	1.22 (0.54, 4.10)	0.68 (0.53, 0.80)	0.20 (0.06, 2.08)	0.50 (0.28, 0.72)
*Anopheles pharoensis*	*	*	0.64 (0.52, 0.83)	17.62 (13.50, 24.00)	2.14 (1.42, 4.66)	3.31 (2.80, 3.92)
*Anopheles tenebrosus*	16.23 (8.28, 46.88)	1.14 (0.82, 1.53)	0.03 (0.00, 0.30)	0.98 (0.36, 2.23)	1.33 (0.84, 2.25)	1.27 (0.70, 2.02)
*Anopheles ziemanni*	10.06 (4.23, 23.90)	1.25 (1.03, 1.45)	0.01 (0.00, 0.08)	1.94 (0.55, 4.98)	0.86 (0.52, 1.61)	1.30 (0.92, 1.75)
*Aedes fryeri*	*	*	1.82 (0.69, 5.77)	1.49 (0.81, 2.49)	4.67 (1.61, 12.30)	1.90 (1.30, 2.69)
*Culex antennatus*	1.42 (0.49, 6.16)	1.44 (1.10, 1.88)	6.59 (0.73, 128.02)	0.44 (0.23, 0.70)	*	*
*Culex pipiens*	0.21 (0.18, 0.24)	2.24 (2.12, 2.38)	0.32 (0.21, 0.56)	1.06 (0.89, 1.23)	0.12 (0.09, 0.16)	1.46 (1.24, 1.71)
*Culex poicilipes*	2.39 (1.41, 4.12)	1.09 (1.04, 1.17)	0.05 (0.04, 0.06)	2.14 (1.83, 2.47)	0.37 (0.25, 0.59)	1.15 (1.01, 1.28)
*Culex quinquefasciatus*	0.13 (0.00, 4.76)	0.28 (0.01, 0.91)	7.31 (0.47, 607.60)	0.35 (0.11, 1.05)	0.01 (0.00, 0.16)	0.42 (0.10, 1.03)
*Culex* * tritaeniorhynchus*	0.57 (0.40, 0.72)	1.94 (1.80, 2.27)	0.13 (0.10, 0.17)	1.96 (1.75, 2.39)	0.33 (0.24, 0.43)	1.78 (1.64, 2.05)
*Mansonia africana*	0.24 (0.18, 0.33)	1.65 (1.43, 1.87)	267.22 (10.53, 11255.65)	0.29 (0.22, 0.40)	0.48 (0.29, 0.99)	1.00 (0.84, 1.18)
*Mansonia uniformis*	3.06 (2.36, 4.33)	0.92 (0.89, 0.95)	1.46 (1.03, 2.28)	0.80 (0.76, 0.85)	0.08 (0.07, 0.09)	1.77 (1.70, 1.83)

*Results unreliable due to sparse data

## Discussion

While the SHK proved a sensitive device for detection of all but the rarest of the full range of species in the study site, it collected far fewer mosquitoes than the HLC caught for most species. This is not an uncommon feature of many traps, and as with the CDC light trap and other traps, does not necessarily mean they are unsuitable for use as epidemiological tools ^48, 52, 53^. Mosquito species detected by SHK and HLC included dominant malaria and arboviruses vector species. Unfortunately, it has yet to be determined how or why the HLC method outperforms numerous traps. The skill of the collectors, often considered a potential limitation of HLC at high densities, did not appear to impair the numbers of individuals captured at the high densities recorded in the present study, while variations in the inherent attractiveness to mosquitoes of different hosts would have influenced performance of the SHK to a similar extent as the HLC. Despite their importance in epidemiological studies, and their decades-long use for surveillance of vector mosquito populations of public health interest, knowledge of how mosquitoes behave at close range to a trap or of how the integration of visual and olfactory stimuli enable mosquitoes to find a stationary host is limited ^54– 57^. Uniquely, the SHK is designed to capture mosquitoes as they descend onto a supine human host in response to the rising body heat and associated attractants, a host-location route previously identified from infra-red tracking studies as the most common route taken by *An. gambiae s.l* and *An. arabiensis* when approaching a human host ^28, 58^. Assuming the SHK was operating as intended, any other species arriving at the host in this way should also have struck the electrocution grid and been killed and captured. This would include *Cx quinquefasciatus* ^37^ but the orientation paths of the other important vector species, have not been characterised. However, horizontal air movements across the host would disperse the plume of attractants and eliminate the focus of attractants at the net roof ^59^ and the SHK would become very ineffective. As such conditions would be common outdoors but rare indoors the SHK would be expected to be more suitable for use indoors than outdoors.

SHK sampling efficiency was, in general, density-dependent suggesting that SHK trap could fail to detect uncommon or rare mosquito species. Several factors, such as trapping location, visual contrast, trap configuration and, as well as climate and surrounding environment features determine trap response of insects during appetitive and attraction flights ^60, 61^. The extent to which climate factors, notably air temperature, relative humidity, and wind speed influence the sampling efficiency of traps still remains poorly known. These factors may influence trap performance by affecting vectors appetitive searching for host clues ^62– 65^, whilst wind speed may directly affect efficiency by reducing or fully discouraging flight activity. Mosquitoes typically fly at a cruising velocity less than 1 m/s so that, wind speed above normal flight speed may completely cease flight activity of several vector species ^66, 67^. During this study air temperature at night was within the optimum range for mosquito flight activity (20°C – 30°C), as reported in another study also conducted in southern Mozambique ^68^ and elsewhere ^63, 69^. Differently, the average wind speed at sunset and, usually at dawn, was around 4.15 m/s, above of what is considered optimum speed for mosquito to fly ^67^.

One of the main advantages of SHK is the fact it is fully human-baited, and in this first evaluation, all the catches comprised unfed malaria and arboviruses vector species, notably *An. arabiensis*, *An. gambiae s.s*, *An. funestus s.s.*, *An. merus*, *An. parensis*, *Cx. quinquefasciatus*, *Cx. tritaeniorhynchus* and *Ma. uniformis*, attesting to its exposure-free. Here, the SHK provided a reliable sample of anthropophilic mosquito species that arrive unfed at the host with a genuine intention of blood-feeding on the volunteer within the bed net. Furthermore, the trap offers an alternative to explore in real time key behavioural components concerning host-finding behaviour by disease vectors using recently developed image-capturing technology such as those reported by Parker *et al*., ^28^. However, it is worth noting that, although both SHK and HLC employ the same source of odour stimuli (natural human host), it is very unlike that they could, thereby, collect absolutely the same quantity of mosquitoes since both are subjected to imprecisions and analytical errors that can generate significant levels of variability ^46, 70, 71^. However, as Hii *et al*., ^48^ have remarked, the lower numbers of mosquitoes caught by an alternative method do not *per se* invalidate the use of the candidate method for estimation of relevant entomological indicators. The crucial criterion is whether the new method is collecting mosquitoes that are in proportion to the gold standard. This condition was fulfilled with *An. gambiae s.l*, *Cx. pipiens s.l* and *Ma. uniformis*, but not with remaining vector species.

One of the key limitations of the SHK trap is dependence on a source of alternating electric current (AC) for operating. However, this limitation could be reduced by installing an AC/DC power convertor or by modifying the electrocution grids to enable them to work with direct current (DC). It was noted that the voltage input would sometimes inflict significant burn damage on mosquitoes, particularly on small-sized mosquitoes such as *An. funestus* rendering them difficult or even impossible to identify morphologically. However, identifying the ideal settings to eliminate this risk may is not a simple or predictable process and finding the optimal level of voltage is likely to be a major challenge. Setting the voltage was compromised by the need to ensure that the live wires comprising the electrocution grid were spaced at a distance to eliminate short-circuiting, but close enough to capture even the smallest anophelines and retaining enough voltage to knock them down, without damaging them beyond recognition.

## Conclusions

In conclusion, the Shockwè trap is a human-baited exposure-free mosquito trapping device with high potential as a sampling device for the indoor and outdoor entomological surveillance of malaria and arbovirus vectors. It also could serve as an experimental device designed to capture mosquito species as they respond to a supine human host. Despite their essential role in elucidation of the mechanism of host location by tsetse, electrocution grids have yet to considered as tools suitable for use in similar studies on mosquitoes ^72^. Structural improvements are possible and could improve trapping at low densities, a challenge for all similar mosquito traps.

## Abbreviations

HLC: Human-landing catch

SHK: Shockwè trap

PCR: Polymerase chain reaction

## Consent

Written informed consent for publication of the participants’ details was obtained from the participants.

## Data Availability

Open Science Framework: Underlying data for ‘The Shockwè trap: a human-baited exposure-free device for surveillance and behaviour studies of anthropophilic vectors’ https://doi.org/10.17605/OSF.IO/6K4MT ^51^ This project contains the following underlying data: Dataset_1: Raw dataset of mosquito species collected using human-landing catch and Shockwè trap in Massavasse village from February to April 2016. Data are available under the terms of the Creative Commons Zero “No rights reserved” data waiver (CC0 1.0 Public domain dedication).
