# Supplementary material for: The Shockwè trap: a human-baited exposure-free device for surveillance and behaviour studies of anthropophilic vectors
Source: Wellcome Open Res. 2023 Oct 12;8:455. [Version 1] doi: 10.12688/wellcomeopenres.19963.1 (PMC11031644; doi:10.12688/wellcomeopenres.19963.1)
Supplement: Peer review history

# Reviewer report: Reviewer response for version 1

**DOI:** 10.21956/wellcomeopenres.22107.r78643

**Published:** 2024-04-19

**Authors:** Emily G McDermott

**Affiliations:** 1 University of Arkansas Fayetteville, Fayetteville, Arkansas, USA

The authors describe the design of a novel trap targeting the collection of anthropophilic mosquito species, particularly *Anopheles*. The SHK trap uses the combination of a human-bait and electric grid to passively collect host-seeking mosquitoes. The SHK trap was compared to human landing catch collections, considered to be the “gold standard” (as the authors state) for studies on human malaria. HLC are not without risk, and are also highly labor and time intensive, and the authors argue that the SHK eliminates many of the drawbacks of HLC. One particular consideration I found interesting was incorporating mosquito behavior to approach sleeping individuals, rather than sitting for HLC, in their trap design.

 The abundance and species composition of collections made indoors and outdoors with both methods was compared. While HLC outperformed the SHK in terms of total numbers of mosquitoes collected of nearly all species, the authors state that the SHK was efficient in sampling the diversity of mosquitoes present at their study site; only three species were caught in the HLC but not SHK, and these species were extremely rare even in the HLC.

 A significant drawback to the SHK is the fact that HLC greatly outperformed SHK for collection of the major human disease vectors, *An. gambiae, Cx. pipiens, *and *Ma. uniformis*. This limitation may pose challenges for the use of this trap for malaria studies, presumably its primary intention. In my view, the authors don’t address this concern in sufficient detail.

 However, there is no such thing as a perfect trap. The benefits of using a device such as the SHK, may outweigh the limitations. I would encourage the authors to discuss what types of studies the SHK would be most appropriate for. There are certainly situations where efficient sampling of species diversity would be preferred over abundance, but the authors should make these considerations explicit. Although outside of the scope of the study, a comparison of *Plasmodium *infection rates in mosquitoes collected by both methods would be a useful metric to determine whether the SHK trap could replace HLC.

 There are a handful of spelling and grammatical errors and typos in the paper which should be addressed.

Is the work clearly and accurately presented and does it cite the current literature?

Yes

If applicable, is the statistical analysis and its interpretation appropriate?

Yes

Are all the source data underlying the results available to ensure full reproducibility?

Yes

Is the study design appropriate and is the work technically sound?

Yes

Are the conclusions drawn adequately supported by the results?

Partly

Are sufficient details of methods and analysis provided to allow replication by others?

Yes

Reviewer Expertise:

Vector biology, vector surveillance

I confirm that I have read this submission and believe that I have an appropriate level of expertise to confirm that it is of an acceptable scientific standard.

# Reviewer report: Reviewer response for version 1

**DOI:** 10.21956/wellcomeopenres.22107.r71027

**Published:** 2024-02-12

**Authors:** Fekadu Massebo

**Affiliations:** 1 Arba Minch University, Department of Biology, Arba Minch, Ethiopia

**Re**: The Shockwè trap: a human-baited exposure-free device for surveillance and behaviour studies of anthropophilic vectors

**General comments**

 The authors presented evidence on the Shockwè trap (SHK). This trap replaces the human land catch method, which poses a risk of human exposure. The authors compared the new trap with HLC against species composition and abundance at the field.

 The authors acknowledged that the HLC method is the gold-standard method for collecting mosquitoes, and they also revealed that there is a higher chance of collectors being infected by mosquito-transmitted pathogens. While it is true that there is a risk of exposure during the HLC, there is evidence suggesting that if the collectors are monitored correctly and provided with prophylaxis, the risk of exposure may not be higher than those not involved in the process, and in some conditions, it may even be lower.

**Specific comments **

- Although the HLC method collected 2.5 times more mosquitoes than the SHK method, the author's conclusion favoured SHK by giving more importance to the number of species collected by each method. Why was the number of species collected preferred over the number of mosquitoes collected for comparison?
- It's worth noting that the SHK trap effectively catches most species of mosquitoes without exposing humans to infection. However, the author did not discuss the inherent limitations of the SHK trap in capturing the most important malaria vector, the *An. gambiae* complex, and its impact on vector surveillance and monitoring for decision-making. Why this point was not discussed?
- The authors did not focus much on discussing the practical implementation of the trap for collecting mosquitoes in rural areas where there is no electricity, despite the fact that mosquito monitoring and surveillance are mainly carried out in these areas. Why wasn't the practicality of SHK traps in malaria-endemic rural areas discussed compared to HLC?
- Why did the authors favour SHK traps despite the limitations in collecting the number of mosquitoes and their practicality in areas that require reliable monitoring tools?
- Additionally, why did the authors tend to SHK traps without concrete evidence indicating that the risk of human exposure is higher during the HLC method? The mosquito control program requires efficient and practical tools to provide suitable data for decision-making with little or no risk of exposure.

Is the work clearly and accurately presented and does it cite the current literature?

Yes

If applicable, is the statistical analysis and its interpretation appropriate?

Yes

Are all the source data underlying the results available to ensure full reproducibility?

Yes

Is the study design appropriate and is the work technically sound?

Yes

Are the conclusions drawn adequately supported by the results?

Partly

Are sufficient details of methods and analysis provided to allow replication by others?

Yes

Reviewer Expertise:

Public health entomology

I confirm that I have read this submission and believe that I have an appropriate level of expertise to confirm that it is of an acceptable scientific standard.
